# 

**Published:** 1924-07

**Authors:** 


### HOSPITAL and HEALTH REVIEW

*Established 1886* *No.* 1876. *Vol. LXXIII*

*New Series.* Vol. III. No. 34. *July,* 1924. Price Sixpence

CONTENTS.

| | PAGE |
|---|---|
| The Search for a Policy | 193 |
| Health of the Empire | 194 |
| Notes and Comments | 195 |
| Mr. Wheatley's Housing Bill—The Mentally Defective—The Cinema in Education—Railway Strikes and Health—An Annual Overhaul—Care of the Consumptive—Hospitals and History—The Seasons and Suicide—The Glasgow Crime—Falling Death Rate from Diabetes—The Unworthiness of Insects—Malaria in America—The Dried Milk Regulations—Home Helps—Tracking down Epidemics of the Typhoid Group | 195 |
| Hospital Men of Mark: Mr. Wade Deacon, C.B.E., and Captain William Rutter | 199 |
| A Syrian Mental Hospital | 200 |
| Tom George: By Miss H. S. Cooper Hodgson | 201 |
| Opening of Biddulph Grange | 203 |
| New Wing of South London Hospital | 203 |
| British Hospitals Association Conference | 204 |
| Voluntary Hospitals and Municipal Authorities. By Dr. Kay Menzies | 205 |
| Approved Societies in Relation to Voluntary Hospitals. By R. J. Meller, M.P. | 207 |
| The Training of Nurses. By Sir Wilmot-Herringham | 208 |
| Sheffield Hospital News | 209 |
| West Park Mental Hospital, Epsom | 209, 213 |
| Extending the Seamen's Hospital | 210 |
| The Bordeaux Conference | 212 |
| The London School of Hygiene | 214 |
| Ice in Hospitals. By Sydney F. Walker | 215 |
| Correspondence | 216 |
| The Voluntary Hospitals Conference | 216 |
| The Healing Waters of Buxton | 217 |
| Hospital Progress and Finance | 218 |
| Reviews of Books | 220 |
| Echoes of the Month | 223 |
| Points from Hospital Reports | 224 |
| Hospital and Health News | 225 |
| Recent Appointments | 226 |
| Answers to Correspondents | 226 |

